# Low desiccation and thermal tolerance constrains a terrestrial amphibian to a rare and disappearing microclimate niche

**DOI:** 10.1093/conphys/coab027

**Published:** 2021-04-28

**Authors:** Emily P Hoffmann, Karen L Cavanough, Nicola J Mitchell

**Affiliations:** 1 School of Biological Sciences, The University of Western Australia, 35 Stirling Highway, Crawley, Western Australia 6009, Australia; 2 Perth Zoo, Department of Biodiversity, Conservation and Attractions, PO Box 489, South Perth, Western Australia 6951, Australia

**Keywords:** Climate change, *Geocrinia*, physiological thresholds, soil moisture, temperature, threatened species

## Abstract

Drier and hotter conditions caused by climate change threaten species that exist close to their physiological limits, as well as those with limited ability to move. Habitat specialists may also be particularly vulnerable if they have specific abiotic requirements. Here we assess whether thermal and hydric constraints can explain the highly restricted and declining distributions of the critically endangered terrestrial-breeding frog, *Geocrinia alba*. We also evaluate the species’ vulnerability to climate change based on the similarity of current microclimatic conditions to their physiological limits. We found that *G. alba* had low thresholds of thermal and desiccation tolerance relative to other anuran species. The estimated thermal optimum (*T_opt_*) and critical thermal maxima (*CT_max_*) were 23.3°C and 29.6°C, respectively, and adult frogs had an absorption threshold (*AT*, the lowest water potential at which water can be absorbed from a substrate) of −50 kPa, the lowest recorded for an amphibian. Comparing environmental conditions and water loss in the field using agar models showed that riparian habitats where frogs occur provide a unique microclimate in the landscape, offering significantly lower desiccation risk during extreme summer conditions compared to immediately adjacent riparian and terrestrial habitats. Monitoring of microclimate conditions within occupied frog habitats over 2 years showed that in extreme dry and hot years the *AT* was exceeded at six of eight sites, and *T_opt_* was exceeded at two of eight sites. Given their specific physiological limits, the apparent rarity of suitable microclimates and a regional drying–warming trend, we suggest that *G. alba* occupies a potentially disappearing niche and may be indicative of other habitat specialists that rely on ephemeral drainages. More broadly, this study highlights that desiccation thresholds may tightly constrain amphibian distributions and need to be considered along with thermal tolerance thresholds when predicting the impacts of climate change.

## Introduction

In a time of rapid global environmental and climatic change it is essential to identify species most at risk of extinction in order to prioritize conservation actions (Sodhi *et al.*, 2008; Pacifici *et al.*, 2015). Amphibians are particularly vulnerable to climate change as key events in their life history are cued by temperature and precipitation (Corn, 2005). Further, as ectotherms, environmental temperatures determine their physiological rates (Rome *et al.*, 1992), and their permeable skins mean they have little resistance to water loss relative to other terrestrial vertebrates (Shoemaker *et al.*, 1992). For amphibians occupying terrestrial environments, soil moisture, and to a lesser extent temperature, plays a crucial role in maintaining their water balance (Spight, 1967), but considerable variation exists in thermal and hydric thresholds across species (Walker and Whitford, 1970; Hillman *et al.*, 2009; Bennett *et al.*, 2018). As critical tolerance levels are unknown for most amphibians (Li *et al.*, 2013), defining the physiological limits of species is crucial for identifying their critical habitat and for predicting their responses to environmental change.

A species’ vulnerability to climate change depends not only on its physiological traits but also on the environmental conditions to which it is exposed (Scheffers *et al.*, 2014). Prediction of a species’ risk can be assessed by investigating the margin between environmental temperatures and physiological thresholds, using metrics such as warming tolerance (WT; the difference between a species’ critical thermal maxima and the mean or maximum environmental temperature) or thermal safety margin (TSM; the difference between a species’ thermal optimum and the environmental temperature) (Deutsch *et al.*, 2008). For example, an ectothermic species at low latitudes may be at high risk due to a narrow TSM or WT (Deutsch *et al.*, 2008; Tewksbury *et al.*, 2008; Sunday *et al.*, 2014). Conversely, temperate species should be well buffered from warming, as they often occupy environments well below their thermal tolerances (Martin and Huey, 2008). However, habitat and climate specialists in temperate areas may have narrow physiological limits, as tolerance ranges are often related to the range of climatic variation of the environment (Janzen, 1967; Addo-Bediako *et al.*, 2000), and some temperate areas may experience appreciable shifts in microclimates with climate change. Mid-latitude areas in particular are predicted to experience among the largest changes in average precipitation and soil moisture, and significant drying is already documented (IPCC, 2014; Seager *et al.*, 2019). Therefore, amphibians that are habitat specialists in temperate regions experiencing changes in temperature and/or precipitation may already be under pressure from climate change.

Here we assess whether thermal and hydric physiological constraints could explain the highly restricted and declining distribution of a critically endangered terrestrial-breeding frog from a temperate Mediterranean climate in southwestern Australia. The white-bellied frog, *Geocrinia alba*, occurs in distinct clusters along drainage lines and headwater streams (Wardell-Johnson and Roberts, 1993) and is strongly associated with areas with cooler and wetter microclimates (Hoffmann *et al.*, 2021). Males show extremely high site fidelity, and there is almost no dispersal between populations (Driscoll, 1997), suggesting they spend their entire life cycle in these habitats. Over half of the known *G. alba* populations have become extinct in recent decades, and extant populations continue to decline (TSSC, 2019). Southwestern Australia, as with other areas with Mediterranean climates, is undergoing a drying trend and has already experienced a 15–20% decline in rainfall and a consequent 35–50% reduction in streamflow since the 1970s (Petrone *et al.*, 2010). Currently, the hydrological and temperature regimes of *G. alba* habitats are unknown, as are the species’ physiological thresholds. Given that the presence and abundance of *G. alba* is closely tied to specific riparian drainages (cooler and wetter sites), we predict that *G. alba* is sensitive to water loss and higher temperatures and consequently is restricted to a narrow microclimate niche in the landscape.

We first estimated thermal and hydric thresholds for *G. alba* and compared them with those of their allopatric sister taxa *G. vitellina*, to assess whether *G. alba* has lower physiological thresholds. *Geocrinia vitellina* shares a similar terrestrial life history and restricted distribution but has not undergone as severe population declines as *G. alba* (Department of Parks and Wildlife, 2015; Hoffmann *et al.,* 2021). We then explored where suitable microclimates exist in the species’ habitat by examining rates of water loss (using agar frog models) and available microclimates in habitats within and surroundings where *G. alba* still occur. Lastly, we compared the estimated physiological thresholds with soil microclimates at a range of *G. alba* sites over 2 years, to determine whether frogs are experiencing conditions near or exceeding their physiological limits. Taken together, these data will help to understand the impacts of current and future environmental changes on the species and to identify critical stress points in the annual life cycle that might trigger mortality and population declines.

## Methods

### Study species and distribution

*Geocrinia alba* is a small (~20 mm) terrestrial-breeding frog that occupies a limited number of forested drainage lines and headwater habitats within a small area (<70 km^2^) of southwestern Australia (−34.09025 S, 115.10904 E) (TSSC, 2019; Wardell-Johnson and Roberts, 1993). The region has a Mediterranean-type climate, with distinct cool–wet winters and dry–warm summers. Frog habitats are well-defined riparian corridors with a dense overstorey and ground layer of rhizomatous vegetation, surrounded by tall open forest dominated by *Eucalyptus marginata* and *Corymbia calophylla* (Wardell-Johnson and Roberts, 1993). The breeding season commences in late winter/early spring (August/September) and continues into summer (December/January) (Driscoll, 1998). Eggs are laid in a shallow depression in moist soil adjacent to ephemeral streams and develop endotrophically until metamorphosis is completed between November and January (Anstis, 2010). *Geocrinia vitellina* is a closely related species with an allopatric distribution, occurring ~6 km to the east.

For both species, data to define thermal thresholds were collected from experiments on frog embryos and larvae, whereas experiments on adult frogs were used to determine hydric thresholds. Animal collections and fieldwork were conducted under permits from the Western Australian Department of Biodiversity, Conservation and Attractions (DBCA), Parks and Wildlife Service (Regulation 17: 08-003026-1; Regulation 15: 11-001286-2; TFA 2019-0058), while all research protocols were approved by the Perth Zoo Animal Ethics Committee (2016-3, 2019-1, 2018-10 and 2019-8) and the University of Western Australia Animal Ethics Committee (RA/3/100/1609).

### Thermal thresholds

#### Estimating temperature thresholds using development rates

Thermal tolerance limits can be approximated using different methods but typically involve exposing organisms to a range of temperatures until a lethal or sub-lethal endpoint is reached (e.g. the onset of muscular spasms or locomotion ceases) (Lutterschmidt and Hutchison, 1997). As we were working with threatened species, we used a non-lethal method to estimate the critical thermal maxima (*CT_max_*) by fitting a thermal reaction norm to data collected on development rates. The fitted function was based on incubation times under constant and fluctuating temperatures, the latter of which aid in approximating development rates at high temperatures that would normally cause mortality if held constant (Dallwitz and Higgins, 1992). We incubated *G. alba* and *G. vitellina* eggs at temperatures naturally experienced in field conditions and recorded the number of days taken between defined developmental stages (see below), which allowed estimation of the temperature at which the development rate peaks (thermal optimum, *T_opt_*) and the upper thermal limit (*CT_max_*).

#### Egg collection and incubation

We excavated freshly laid *G. alba* (*n =* 9) and *G. vitellina* (*n* = 13) egg clutches from the field (stages 7–19; Gosner, 1960) during the breeding seasons (September–October) of 2018 and 2019. Egg clutches were divided into smaller groups of four to eight eggs (split clutches) and placed in individual containers filled with moist soil collected from the breeding sites. Eggs were then transported to Perth Zoo within 72 hours of collection where each split clutch was randomly allocated to an incubation treatment of either constant (~15, 18, 20, 21, 25°C) or fluctuating (21 ± 2.5°C, 25 ± 5°C) temperatures (Table S1; Fig. S1) (Steridium, models i140 and i500). The fluctuating temperatures were programmed to follow a sine-wave cycle that mimicked diel temperature fluctuations experienced in the field (S. Conroy unpublished data). A temperature logger (Thermochron iButton DS1921H-F5 or HOBO UA-001-08) was placed in each container adjacent to the embryos to record hourly incubation temperatures for each split clutch. Each split clutch was kept moist by spraying reverse-osmosis (RO) water on its surface and was monitored daily.

Development times were calculated as the average number of days from stage 19 to stage 39 according to developmental stages defined in Anstis (2010) for *Geocrinia* species. Stage 19 (hind limb buds just visible) was the earliest stage that all embryos were collected at, and stage 39 was just prior to metamorphosis (tail stub < 2 mm), when metamorphs are routinely transferred to different terraria, according to Perth Zoo husbandry practices.

#### Estimation of *Topt* and *CTmax*

The effect of incubation temperature and species on development time was analysed using a linear mixed-effect model, with clutch origin included as a random factor. However, as the random effect was not significant (*P* = 0.56), a linear model and analysis of variance (ANOVA) were used.

A non-linear development rate function was fitted for *G. alba* and *G. vitellina* larvae using the program DEVARA (Dallwitz and Higgins, 1992), which accommodates both constant and variable temperature regimes. We entered average development times (number of days between stage 19 to 39) for each split clutch with their average temperature (constant temperatures) or hourly temperatures (fluctuating temperature treatments) based on recordings from the temperature loggers. We also included development times for *G. vitellina* at 15°C (calculated for the same developmental period—stages 19–39) from Mitchell (2001a).

The non-linear development rate function is defined by five parameters (see Supplementary Material). The temperature at which the development rate peaks (i.e. the thermal optima, *T_opt_*) for each species was assumed to be parameter *b_3_*. The *CT_max_* is not an estimated parameter, but we approximated this from the temperature where the development rate was predicted to fall to 0 for each species (Ruiz-Aravena *et al.*, 2014). While thermal tolerances can vary according to life stage and acclimation temperature, in amphibians, the *CT_max_* of larvae is often higher than that for juveniles and adults but generally varies <1–2°C between life stages (Krakauer, 1970; Delson and Whitford, 1973; Cupp, 1980; Sherman and Levitis, 2003; Beltrán *et al.*, 2019; Enriquez-Urzelai *et al.*, 2019). Likewise, *CT_max_* can vary with acclimation temperature, but on a similar scale of 1–2°C (Krakauer, 1970; Gvoždík *et al.*, 2007; Oyamaguchi *et al.*, 2018; Enriquez-Urzelai *et al.*, 2019). Therefore, we considered our thermal sensitivity estimates based on embryonic and larval development to be informative for assessing thermal stress across all *G. alba* life stages.

### Water absorption thresholds

To determine hydric thresholds for *G. alba* and *G. vitellina* we assessed rates of water loss on a range of wetted substrates to determine the absorption threshold—the water potential of a substrate above which an individual gains water, and below which loses water (Heatwole and Lim, 1961). A total of 10 *G. alba* and 10 *G. vitellina* adult males were collected from the field in November 2018 and housed at The University of Western Australia in a controlled temperature room at 18°C with a 13.5/10.5 hour light/dark photoperiod to mimic spring conditions. Males were acclimated for 1 week prior to experiments and fed a diet of pinhead crickets (*Acheta domestica*) but were not fed 3 days prior to experiments to reduce the chance of defecation. Experiments were conducted at ~18°C and 65% relative humidity.

#### Water balance experiments

Frogs were kept in very moist terraria (on saturated soil and moss) and were placed in 90-mm petri dishes with 20-ml distilled water an hour prior to the experiment to ensure full hydration. Frogs were washed free of any soil or moss and induced to urinate using a glass cannula gently inserted into the cloaca. They were then blotted with paper towel and weighed to the nearest 0.001 g with a SHIMADZU UW620H calibrated balance to determine their standard body mass.

Frogs were then dehydrated to ~90% of their standard body mass by placing each frog in a small enclosure (a PVC ring wrapped in nylon mesh) within a desiccator, suspended on a mesh platform above ~500 g of Drierite (W.A. Hammond Drierite Co.). Humidity inside the desiccator was between 20% and 30%. The enclosures provided a confined dark environment to reduce stress and movement and allowed easy handling of the frogs for weighing. Frogs were weighed intermittently (approximately every hour) and then more frequently when approaching ~90% of their standard mass.

To determine the absorption threshold, frogs dehydrated to ~90% of their standard mass were placed individually on wetted stacks of 90-mm Whatman 40 filter paper in 90-mm glass petri dishes. To create a range of desired water tensions (0 to −200 kPa), we had first added the amount of water needed, per unit of dry paper weight, based on the established water content curve for Whatman paper 42 (ATSM 5298-10, 2010), and the dishes were sealed with electrical tape. Filter papers were left to equilibrate for 1 week (based on pilot experiments) to make sure water spread evenly through the stack. Dishes were weighed once during the week and topped up with water if necessary and were weighed again before the experiments to validate the water potentials. The gravimetric water content (% water to % paper weight) of treatments ranged from 350% (0 kPa) to 39% (−200 kPa).

Frogs placed on filter paper stacks were re-weighed after 2 hours to determine their rate of water uptake or loss and then immediately rehydrated on filter papers flooded with 20-ml distilled water, which covered their entire ventral surface. Frogs were blotted on paper towel and weighed intermittently (approximately every hour, more frequently when approaching 100%) until they regained 100% of their standard body mass. Then, to refine an initial estimate of the absorption threshold, we conducted a second experiment using the same methodology, but with only two water potential treatments. We selected two water potentials that respectively produced minimal water uptake and water loss from the previous experiment (−50 kPa and −70 kPa), which indicated these treatments were close to the absorption threshold. The same 10 frogs of each species were randomly allocated to one of the two treatments, weighed after 2 hours, and the rate of water uptake was calculated as above. Frogs were rested for 7 days between the two experiments, and any frog that defecated or urinated during either experiment was excluded from the analysis.

Dehydration and rehydration rates were compared between species using an analysis of covariance (ANCOVA) with standard mass as a covariate. The rate of water uptake at different substrate water potentials was calculated as the mass of water gained or lost over the 2-hourly period, converted to rate per hour per unit surface area of the frog (mg cm^−2^ h^−1^). The surface area of each frog was estimated using an empirical equation based on body mass: surface area = 9.9*mass^0.56^ (McClanahan and Baldwin, 1969).

### Water loss and microclimates across riparian and terrestrial habitats

#### Model development and validation

To evaluate spatial patterns of water loss in the field we constructed models made of agar that lost water at a comparable rate to that of live frogs (e.g. Spotila and Berman, 1976; Schwarzkopf and Alford, 1996; Navas and Araujo, 2000). Using a resin frog replica of a similar profile to *Geocrinia* spp*.*, we produced a silicone mould of adult- and juvenile-sized frogs and constructed models of frogs made of 3% agar. To validate that models lost water at similar rates to live frogs, we compared the dehydration rates of models made of 3% agar with that of the 20 live adult male frogs (10 *G. alba* and 10 *G. vitellina*) used in the absorption threshold experiments. We placed 10 adult sized agar models into the same individual mesh enclosures within desiccators with ~500 g Drierite, as was the process for live frogs (see above). Models were weighed at regular intervals until they reached 90% of their initial mass (as for live frogs) and the temperature and humidity were the same as for the frog experiments.

Agar frog models dehydrated at similar rates to live frogs when accounting for their initial mass (slope: *F*_2,24_ = 0.23, *P* = 0.80; intercept: *F*_2,26_ = 0.35, *P* = 0.71) (Fig. S2); therefore, agar models were considered valid proxies for investigating water loss of frogs in field environments. For subsequent field experiments (see below) we refined the adult models to the size of a large *Geocrinia* adult (1.97 ± 0.16 g) and juvenile models were the size of a large 14-month-old frog (0.46 ± 0.07 g) (Perth Zoo, unpublished data), in order to more realistically represent actual frogs.

#### Field rates of water loss and microclimate conditions

Field experiments were conducted during summer and autumn months, which are the driest and hottest time of year when frogs are likely to experience the most extreme conditions. We conducted experiments in autumn 2019 (March 27–28) during mild–dry conditions and in summer 2020 (February 3–4) during extremely hot–dry conditions (maximum daily air temperatures of 24.5°C and 34.1°C for each period, respectively). In 2019 we only used adult-sized models and in 2020 we used both adult- and juvenile-sized models. Experiments were conducted at three to five sites where *G. alba* populations persist and where the extent of populations (based on the distribution of calling males) has been monitored and mapped along riparian drainage lines (DBCA, unpublished data). Sites were also chosen that were in relatively close proximity to each other so they could be sampled on the same day.

#### Water loss across ground cover and habitat types

To investigate whether different ground cover provides different microclimates and protection against water loss we placed agar models under four available ground cover types: leaf litter, moss, sedge and log, and in the open with no cover (control). We placed two to three replicate models per cover type within riparian habitats at three different sites where *G. alba* populations occur.

To evaluate whether water loss varied spatially across habitats we placed models in three different habitat types at five sites. At each site we placed models in riparian drainage habitats where frogs are known to occur (frog habitat), in directly adjacent riparian drainage habitats (adjacent riparian habitat) and in adjacent terrestrial vegetation (adjacent terrestrial habitat). We placed frog models along transects at three distances from the edge of the extent of where frog populations occur (0 m, 25 m, 50 m) into each habitat type. All models were placed directly on the soil underneath leaf litter, which was a groundcover present across all habitat types and was shown to provide good protection from desiccation (see below).

For both experiments, we deployed models during daylight hours to record rates of water loss during times of highest desiccation risk. Pre-weighed agar models (to 0.001 g) were placed within 2 hours of sunrise and collected within 2 hours of sunset and were then re-weighed to measure water loss. Models were transported from a field station to and from field sites in small plastic sealed containers inside an insulated box to minimize condensation. We recorded the time deployed and collected for each site and calculated the percent mass loss per hour that models were deployed.

During each deployment, a temperature logger (Thermochron iButton DS1921H-F5 or HOBO UA-001-08) was placed directly next to each agar model and recorded temperatures hourly. Soil moisture [volumetric water content (VWC), in %] was also measured where models were placed in each habitat type (frog habitat, adjacent riparian, adjacent terrestrial) with a hand-held moisture meter (ICT International, MP306). As the water available to organisms is determined by the soil water potential, a measure that is independent of soil physical properties, we estimated the corresponding range of soil water potentials for each measure of VWC using soil water retention curves previously determined in the laboratory (see Supplementary Material).

#### Statistical analysis

To validate the use of agar models, we compared the rate of water loss of the models to that of live frogs using ANCOVA, with body mass as a covariate. As there were some minor differences in the time models were deployed and retrieved, we calculated water loss per hour and then standardized daytime exposure to a 9-hour period (the minimum time which all models were exposed). To compare water loss across habitats we used models only from 25 m and 50 m distances, as models at 0 m were all located at the edge of the habitat boundaries. Comparisons of water loss, temperature, soil moisture or groundcover type versus habitat type and sampling season were made using ANOVAs and *post-hoc* comparisons were made using Tukey’s honest significant difference (HSD) tests. Temperatures in frog riparian habitats for each sampling season were compared using a *t*-test. Statistical analyses were carried out in R (R Core Team, 2019).

### Seasonal changes in microclimates in frog habitats

To investigate microclimate conditions in frog habitats throughout the year, we installed MPS-6 soil water potential and temperature sensors (Decagon Devices, Inc.) at eight *G. alba* sites. We selected sites across the species’ range that spanned a broad range of soil moisture and temperature values recorded in autumn 2018 (Hoffmann *et al.*, 2021). Sensors were placed 2 m into the riparian vegetation from the edge of the terrestrial-riparian zone vegetation. At one site, a sensor was also installed 2 m into the adjacent terrestrial vegetation. Sensors were installed vertically in the top layer of the soil with the ceramic water potential sensor sitting ~100 mm below the soil surface and temperature sensor ~50 mm below the soil surface. Sensors were programmed to record soil water potential and soil temperature every 30 min for 1–2 years (between July 2018 and June 2020) using Em50 data loggers (METER Group, Inc.) The sensor positions represented a ‘good refuge’ scenario as they recorded the coolest microclimates available when compared to the range of microhabitats sampled within frog habitats (Fig. S3).

#### Statistical analysis of microclimates versus physiological thresholds

The proportion of days exceeding *T_opt_*, *CT_max_* or *AT* was calculated for each site over a 12-month period (July–June). The correlation between the number of days that soil water potentials exceeded the estimated absorption threshold in 2019–20, and the estimated population size of each site was tested using Spearman rank-order correlation. Population estimates were based on the number of calling male frogs at a site (as per Hoffmann *et al.,* 2021) and assuming a 50:50 sex ratio (Driscoll, 1996). WT was calculated as the difference between the estimated thermal maxima (*CT_max_)* and the maximum habitat (soil) temperature (*T_hab-max_*).

### Trend analysis of the recent climate

To examine recent climate trends in the study region we obtained local weather station climate and stream flow data within 25 km of *G. alba* populations (Table S4). Variables analysed included annual mean maximum air temperature (1926–2019), total rainfall (1897–2019) and mean streamflow discharge (1995–2019).

For each variable we used rank-based nonparametric Mann–Kendall trend tests and Sen’s slope estimates to detect the magnitude and significance of trends across all years, and over the past 30 years (where data were available), using the ‘trend’ and ‘Kendall’ packages in R (McLeod, 2011; Pohlert, 2020). Each time series was examined for autocorrelation and autocovariance using ‘acf’ function in ‘stats’, which showed there were no significant cyclical patterns. To examine if the weather during the study years was typical, we also calculated temperature and rainfall anomalies for each year as the deviation from the recent climate mean. We used the average of years 1961–1990 as the recent climate mean, which is defined as the current international standard reference period for calculating climate normals by the World Meteorological Organization (WMO, 2017).

## Results

### Thermal thresholds

**Figure 1:**
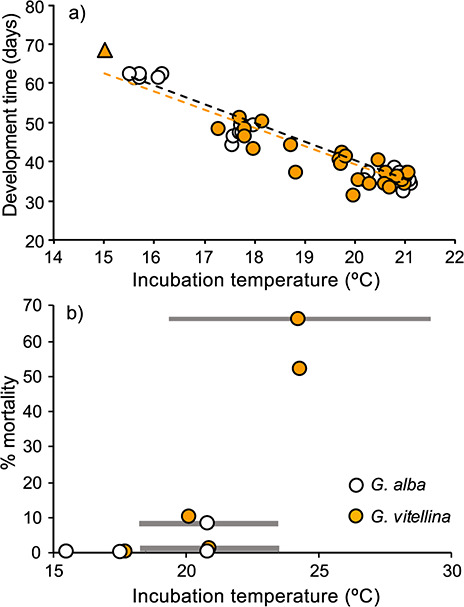
Development times (a) (average number of days between stages 19–39) and rates of embryonic mortality (b) during the initial 5 days of incubation of *Geocrinia alba* and *Geocrinia vitellina* at various temperatures. Points show average temperatures for each clutch; grey bars show the fluctuating temperature range; triangle denotes a data point derived from Mitchell (2001a)

Temperature had a significant effect on the developmental time of larvae (from stages 19–39), which ranged from 36 and 35 days at 21°C to 63 and 69 days at 15°C for *G. alba* and *G. vitellina*, respectively (Fig. 1a) (ANOVA, *F*_(2,48)_ = 224.7, *P* < 0.001). *Geocrinia alba* and *G. vitellina* did not differ in their development rates (*P* = 0.20).

**Figure 2:**
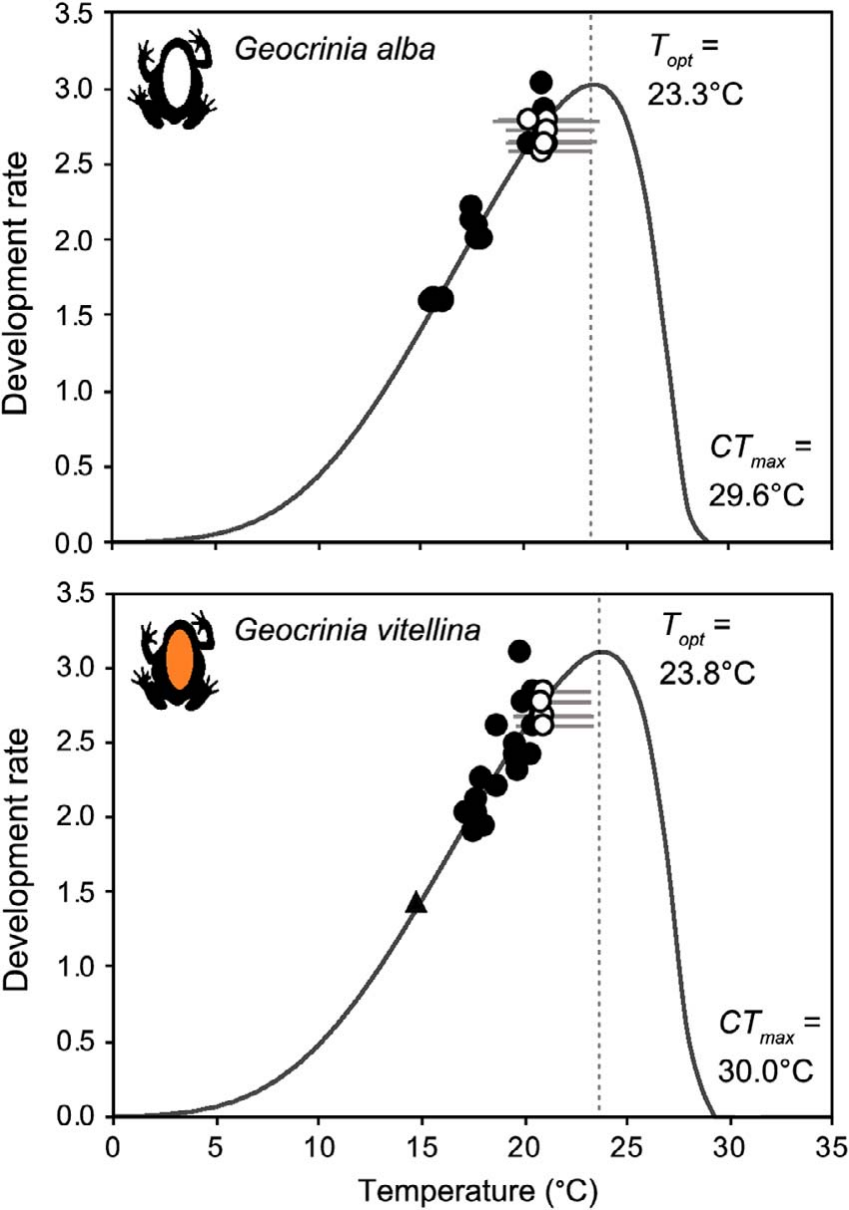
Fitted development rate functions for *Geocrinia alba* and *Geocrinia vitellina*, with each point representing mean rates for two to eight individuals at the specified averaged temperature. Filled circles are data from constant incubation regimes, and open circles are fluctuating temperature regimes with grey bars showing the average daily temperature range. Dashed lines indicate the estimated thermal optimum (*T_opt_*) and *CT_max_* is the upper temperature where the rate equals zero. Parameter values (b_1–5_) for each species are shown in Table 1

Fitting development rate functions using constant and fluctuating incubation temperatures produced similar estimates of peak development rates (*T_opt_*) for *G. alba* and *G. vitellina*, at 23.3°C (±0.7) and 23.8°C (±2.4), respectively (Fig. 2, Table 1). The estimated *CT_max_* was also similar, at 29.6°C for *G. alba* and 30.0°C for *G. vitellina*. *Geocrinia vitellina* embryos reared at 25°C and 25 ± 5°C experienced high larval mortality after 5 days of incubation (Fig. 1b), suggesting temperatures >23.8°C were sub-optimal (Table 1). When temperature treatments were lowered to 21°C and 21 ± 2°C, ≥90% percent of remaining tadpoles survived. Mortality rates at all other cooler treatments were low (<10%) (Fig. 1b).

**Table 1:** Estimated parameters (±SE) from fitted thermal development rate curves for *Geocrinia alba* and *Geocrinia vitellina*

	RSS	*b_1_*	*b_2_*	*b_3_ (T_(opt)_)*	*b_4_*	*b_5_*	CTmax
*Geocrinia alba*	18.91	3.02 (0.11)	8.94 (0.72)	23.33 (0.70)	4.9 (5.50)	0.4 (fixed)	29.6
*Geocrinia vitellina*	43.28	3.10 (0.43)	8.76 (3.60)	23.80 (2.40)	5.37 (23.60)	0.4 (fixed)	30.0

RSS = residual sum of squares

### Water absorption thresholds

**Figure 3:**
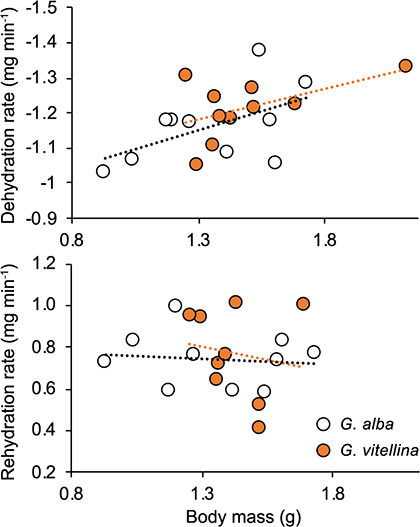
Dehydration rates (top) and rehydration rates (bottom) for *Geocrinia alba* and *Geocrinia vitellina*. Dehydration rates are from 100% to 90% of standard body mass, and rehydration rates are from 89(±2) to 100% standard body mass.

Frogs weighed 1.35 ± 0.27 g (*G. alba*; *n* = 10) and 1.49 ± 0.26 g (*G. vitellina*; *n* = 10) before being dehydrated to 90% of their standard body mass. Rates of dehydration and rehydration did not differ between species (dehydration, *F*_(1,16)_ = 0.29, *P* = 0.59; rehydration, *F*_(1, 15)_ = 0.20, *P* = 0.67) (Fig. 3). Dehydration rate had a significant positive relationship with body mass (*F*_(1,16)_ = 6.14, *P* = 0.02), but rehydration rate did not (*F*_(1, 15)_ = 0.20, *P* = 0.67).

**Figure 4:**
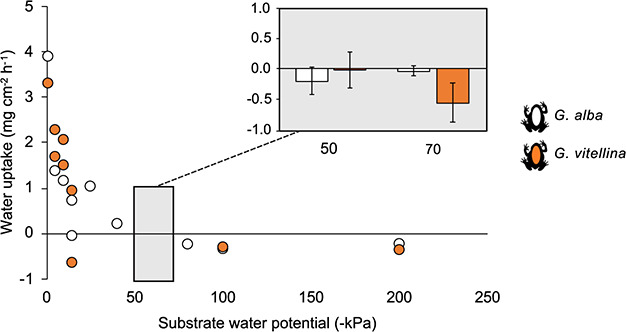
Rate of water uptake as a function of substrate water potential for *Geocrinia alba* and *Geocrinia vitellina.* Circles represent rates for an individual frog (*G. alba*, *n* = 10; *G. vitellina*, *n* = 9). Inset shows the average water uptake (±SE) for five frogs resting on substrates wetted to −50 and −70 kPa. The horizontal line at zero denotes no water loss or gain (higher values indicate frogs gained water, lower values indicate frogs lost water).

The rate of water uptake was highest in water (0 kPa) and dropped rapidly between 0 kPa and −15 kPa for *G. alba* and *G. vitellina* (Fig. 4). Water uptake was possible on substrates with water potentials wetter than −40 kPa, but from −80 kPa all individuals of both species lost water. The second experiment further indicated that at water potentials of −50 and −70 kPa frogs of both species could not rehydrate (Fig. 4, inset). Therefore, the estimated absorption threshold (where both species neither gained nor lost water) for *G. alba* and *G. vitellina* is approximately −50 kPa. The equivalent soil moisture value across *G. alba* and *G. vitellina* sites depends on soil type and ranges from 10.5% VWC in sandy soil to 27.6% VWC in clayey soil (Fig. S5).

**Figure 5:**
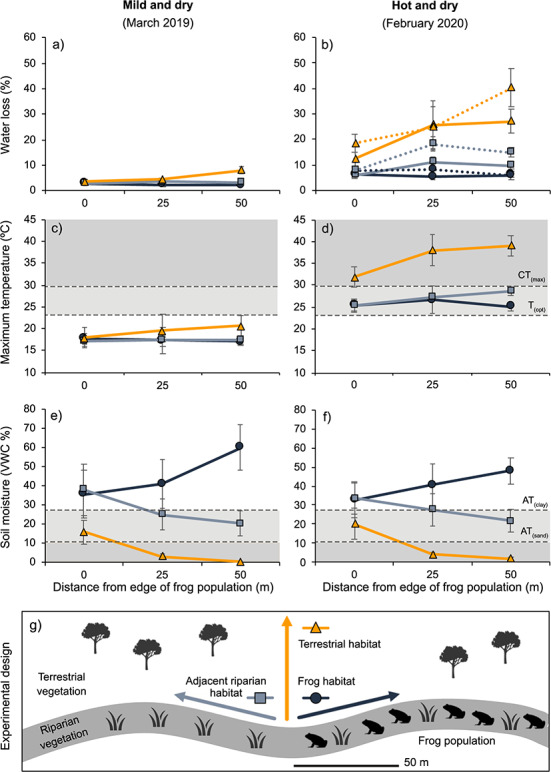
Daytime water loss (±SE) in agar frog models (a–b), soil temperature (c–d) and soil moisture (e–f) along transects in frog habitat, adjacent riparian habitat or adjacent terrestrial habitat. Agar models and temperature loggers were placed at 0 m, 25 m and 50 m in each habitat type (g)*.* All frog models and temperature sensors were placed underneath leaf litter, and water loss was standardized for a period of 9 daylight hours. Dotted lines in panel b represent juvenile sized models. Light and dark grey shaded areas in panels c–f indicate physiological thresholds for *Geocrinia alba*, showing values above the thermal optimum (*T_opt_*) and thermal maximum (*CT_max_*) (c–d), and below the absorption threshold across clay (*AT_clay_*) and sandy soils (*AT_sand_*) (e–f)

### Spatial variation in water loss and microclimates

#### Water loss and microclimates across riparian and terrestrial habitats

Water loss during the summer was significantly lower in riparian habitat where frogs occurred compared to adjacent terrestrial habitat, as well as to adjacent riparian habitat (ANOVA, *F*_2,79_ = 37.39, *P* < 0.001, Tukey’s HSD, *P* < 0.05) (Fig. 5a and b). The difference between habitats was greatest during hot conditions in summer 2020 when agar models lost water at a significantly higher rate (*F*_1,79_ = 54.40, *P* < 0.001). Frog models lost an average of 6.4% water in frog habitat, compared with 13.5% in adjacent riparian habitat and 31.0% in adjacent terrestrial habitat (at 25–50 m sites). Across all habitat areas, juvenile models lost significantly more water (33.9%) than adult models (*F*_1,79_ = 7.54, *P* = 0.008).

Temperature and soil moisture also varied significantly across habitats (temperature: *F*_2,83_ = 49.99, *P* < 0.001; soil moisture: *F*_2,83_ = 49.99, *P* < 0.001). Adjacent terrestrial habitats were hotter and drier compared to riparian habitats across both sampling periods (Fig. 5c--f). Riparian habitats where frogs occur had the highest moisture content (Tukey HSD, *P* < 0.001) and the lowest temperatures in both sampling events (although temperature was not significantly different; Tukey HSD, *P* > 0.05).

Comparing the observed temperature across habitats with temperature thresholds of *G. alba*, terrestrial habitats recorded maximum temperatures up to 9°C above *G. alba CT_max_* in summer 2020, and both riparian habitats recorded temperatures above *T_opt_* (but below the *CT_max_*) (Fig. 5c and d). Soil moisture in terrestrial habitats fell below the absorption threshold in both years (Fig. 5e and f). Adjacent riparian habitats were potentially exceeding the absorption threshold at 50 m from the edge of frog habitats, depending on the soil type.

#### Water loss under different ground cover types

Within riparian frog habitats, water loss was significantly lower under all ground cover types compared with the open control (Fig. S4a and b) (ANOVA, *F*_4,76_ = 26.32, *P* < 0.001; Tukey’s HSD, *P* < 0.001). Agar models lost between 3% and 9% of water under cover, compared with 23–36% water loss with no ground cover. There was no significant difference between water loss under litter, log, moss or sedge cover (Tukey’s HSD, *P* > 0.05). Temperature was also significantly lower under all ground cover types compared to open sites (ANOVA, *F*_4,84_ = 22.93, *P* < 0.001; Tukey HSD, *P* < 0.001), and up to 15.1°C cooler in 2020 than the control models left in the open (Fig. S4c and d).

Agar models of juvenile frogs generally lost water at higher rates compared to adult models, losing up to 36% of their mass in 9 hours without cover, but the difference was not statistically significant (ANOVA, *F*_1,76_ = 3.41, *P* = 0.07) (Fig. S4b). Water loss was higher in February 2020 (ANOVA, *F*_(1,76)_ = 23.41, *P* < 0.0001) when habitat temperatures were significantly warmer (ANOVA, *F*_(1,84)_ = 39.70, *P* < 0.001); (Fig. S4c and d).

### Microclimates in frog habitats over time and physiological tolerances

**Figure 6:**
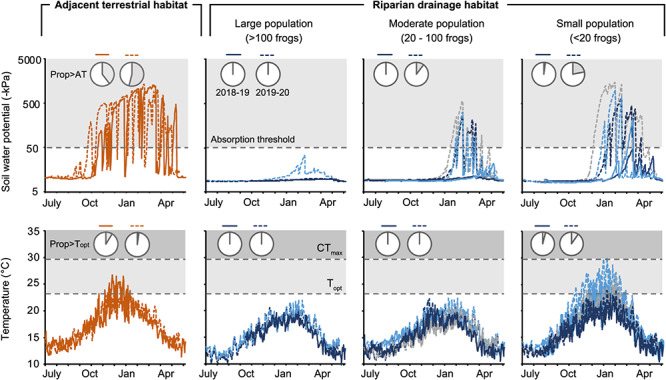
Soil microclimates (water potential and temperature) at *Geocrinia alba* riparian sites and adjacent terrestrial habitat in 2018–19 (solid lines) and 2019–20 (dashed lines) and estimated thermal (*Topt* and *CTmax*) and hydric (*AT*) tolerances (grey dotted lines). Pie charts show the average proportion (Prop) of days exceeding *AT* (top row) or *T_opt_* (bottom row) in each year. Riparian sites are grouped by large (*n* = 2), medium (*n =* 3) and small (*n =* 3) population size, based on population estimates in 2017–18. *AT*—absorption threshold, *T_opt_*—thermal optimum, *CT_max_—*thermal maximum

Soil water potential varied considerably across *G. alba* sites and between years (Fig. 6). Soils at all sites were saturated (generally > −10 kPa) over the wet–cool winter months, but dried to different extents during the drier–hotter months. In 2018–19 soils dried between February and May and one riparian frog site exhibited soil water potentials that were drier than the absorption threshold (*AT*). In 2019–2020 soils were drier overall and most (six of eight) *G. alba* sites recorded soil water potentials drier than the absorption threshold (Fig. 6). The number of days that soil water potentials were drier than the absorption threshold was negatively correlated with the estimated population size (*r_s_ =* −0.79, *P* = 0.02). Soil water potentials in adjacent terrestrial habitat were much lower than the absorption threshold for the greatest proportion of the year and were only wetter than the *AT* between June and August (Fig. 6).

Soil temperatures at *G. alba* sites generally ranged between 10°C and 20°C; however, three of eight sites exceeded the *T_opt_* (range, 3–86 days) between November and March and one site exceeded the *CT_max_* on 2 days (Fig. 6). Sites that experienced temperatures exceeding *T_opt_* and *CT_max_* were those with the smallest populations (<20 frogs). The current average WT across *G. alba* habitats (*CT_max_—T_hab-max_*) was 6.4°C but ranged from 9.3°C to −0.4°C. Comparing the mean number of days exceeding thresholds across all sampled *G. alba* sites, the absorption threshold was exceeded more frequently (26.2 days) compared with thermal tolerances (*T_opt_*) (9.6 days).

### Trends in the recent climate

Climate trend analysis showed that rainfall near *G. alba* sites has decreased significantly over the past 30 years (−109 mm decade^−1^), equating to a 29.2% decline in rainfall compared to the recent (1961–1990) average (Table S4, Fig. S6). Annual streamflow discharge from 1995–2020 at Chapman Brook showed a 36.7% reduction in streamflow over the 24-year record period, but the negative trend was not significant. Annual mean maximum temperatures have increased significantly over time, rising by +0.13°C decade^−1^ over the past 30 years (Table S4; Fig. S6).

The rainfall during the years of the study was below average in both years (Table S5). The second year, 2019, was the second driest year on record (1926–2019) and total streamflow was 52.4% lower than average (1995–2019) (Table S5). Mean maximum temperature in both years was above average and was greater than the 80th percentile (Table S5).

## Discussion

Here we determined physiological thresholds in the context of the contemporary microclimatic niche and identified a threatened species at immediate risk of physiological stress due to occupancy of unsuitable abiotic environments. We further demonstrated that *G. alba* is constrained to a rare and possibly disappearing microclimate niche, which provides a mechanistic explanation for their recent and ongoing declines.

### Hydric and thermal thresholds

*Geocrinia alba* and their sister taxa *G. vitellina* have low physiological thresholds compared to other amphibians. Absorption thresholds have only been determined for a small number of species but in anurans range from approximately −80 to −1000 kPa (Walker and Whitford, 1970; Cartledge *et al.*, 2006) and in salamanders from approximately −100 to −300 kPa (Heatwole and Lim, 1961; Spight, 1967; summarized in Hillman et al., 2009). Hence, the absorption threshold of *G. alba* and *G. vitellina* of −50 kPa is the lowest recorded for any amphibian species and indicates extreme sensitivity to losing water to dry substrates. Further, neither species showed any cutaneous resistance to water loss, based on their similar rates of water loss to agar models. From a thermal perspective, the *CT_max_* of *G. alba* and *G. vitellina* are also low (29.6°C and 30°C, respectively), ranking seventh and ninth lowest of estimates for 114 anurans in a global database of thermal tolerances (GlobTherm; Bennett *et al.*, 2018). Other species with low *CT_max_* include Australian terrestrial breeding frogs, such as *Cophixalus* species, which are constrained to narrow ranges in tropical montane regions of northern Queensland (*CT_max_* 28.09–32.77°C; Storlie *et al.*, 2014) and three *Philora* species that occur in boggy seepages at high temperate elevations in New South Wales (*CT_max_* 24.8–30.5; Brattstrom, 1970). Together, the low absorption threshold, no resistance to water loss and low *CT_max_* support our hypothesis that both species require stable moist and cool habitats.

### Available microclimates and potential habitats

The physiological constraints we have identified for *G. alba* offer a compelling explanation for its restricted distribution and low tendency to disperse between habitat patches. Specifically, we suspect that the species is restricted to sections of riparian habitat that provide a specific microclimate of cool temperatures and high soil moisture, notably during the drier non-breeding season. Groundcover within these habitats provides a significant buffer against extreme temperatures and evaporative water loss on the warmest and driest days in summer and autumn. Protection from water loss appears particularly important for juvenile frogs, as models were shown to have higher water loss compared to adult-sized models, reflecting their higher surface area to volume ratio (Tracy, 1976). In contrast, the adjacent terrestrial habitats seem uninhabitable for most of the year, as evaporative water loss of agar models was high and both soil temperatures and soil dryness exceeded the estimated physiological thresholds measured here. Driscoll (1997) studied the movement of *G. alba*, and while most adults remained entirely within the riparian habitat, a limited number of frogs were observed moving out of riparian drainage sites in late autumn into adjacent terrestrial habitat and returning in late winter before the breeding season. This coincides with when terrestrial habitats were shown to be significantly cooler and wetter (soils were saturated) in our study and provides suitable microclimates that would allow frogs to utilize these habitats. However, dispersal across terrestrial habitats is very unlikely outside of these wet and cool periods, and we speculate that *G. alba* is largely limited to dispersing along riparian corridors.

### Frogs and current climate extremes

Our study revealed that the current soil microclimates at frog sites periodically breach *G. alba*’s physiological thresholds. In 2018–19, soil microclimates within riparian frog habitats were sufficiently cool and moist throughout the dry summer months, except briefly at one site. Conversely, in 2019–2020, the weather was significantly drier and hotter, and soils at most frog sites dried to water potentials that exceeded the *Geocrinia AT* of −50 kPa. Moreover, three of those sites also recorded temperatures exceeding *T_opt_*, with one of those breaching the estimated *CT_max_* (Fig. 6). The actual conditions an individual frog is exposed to can be significantly buffered by behaviour and microclimate shelter selection (Seebacher and Alford, 2002; Scheffers *et al.*, 2014). However, our microclimate measurements very likely represented a ‘good refuge’ scenario, as we profiled soil microclimates that represented the most buffered microhabitat conditions (Fig. S3). We also sampled within the core areas of frog populations, which we showed were cooler, moister and hence provided better protection against water loss than surrounding riparian and terrestrial habitats. The actual temperatures frogs experience are potentially lower, as we measured environmental temperatures, not body temperatures, which could be slightly cooler due to evaporative water loss (Rome *et al.*, 1992). Likewise, we assumed that *G. alba* does not burrow or have access to deeper soil layers that may be cooler or wetter. We did, however, explore spatial variation in microclimates across the species’ range by sampling a number of populations, revealing considerable microclimate variation across sites, despite the small distances between populations (0.65 to 11 km). The cumulative breaches of physiological thresholds were correlated with frog population size, with small populations (<20 calling males) occurring in areas with the most stressful hydric and thermal conditions. Thus, it seems likely that rapid warming and drying due to climate change, and associated extreme events such as heatwaves, pose a critical immediate threat across the species range and provide a mechanism that explains the observed declines and population extinctions of *G. alba*. A priority should be to examine soil microclimates in the habitats occupied by *G. vitellina*, which has one recorded population extinction, to determine whether they are experiencing similar climatic pressures (Department of Parks and Wildlife, 2015).

The heterogeneity in microclimates across seemingly analogous habitats occupied by *G. alba* reiterates the importance of considering microclimates and climatic extremes across a number of areas within a species range (even for species with small ranges) when making predictions about impacts from climate change (Gerick *et al.*, 2014). Here, by capturing hourly data on microclimate conditions across various habitats within *G. alba*’s range, we have identified only two sites that remained suitable year round and between years, never breaching the species’ thermal or hydric thresholds. These sites currently support two of the largest *G. alba* populations, which is likely due to their highly stable and favourable conditions, and hence should be defined and protected as critical habitat for the species. Over the 2 years surveyed, these areas had a WT (*CT_max_ − T_hab-max_*) of 7.5–8.4°C, and a soil water potential buffer of −16 to −41 kPa before reaching potentially stressful conditions. Monitoring the microclimates at these two sites could be a key indicator for triggering conservation actions for the species, such as whether to consider assisted colonization to new areas outside of their natural range (Rout *et al.*, 2013).

### A disappearing niche?

Given their physiological limits, the apparent rarity of suitable microclimates and the current drying–warming trend in southwestern Australia, we suggest that *G. alba* occupies a disappearing microclimatic niche. An analogous situation is experienced by montane species that are constrained to narrow and isolated climatic niches that are predicted to contract upward in elevation as the climate warms, potentially leading to extinction (Peters and Darling, 1985; Lemckert and Penman, 2012; Lyons and Kozak, 2020). Here we suggest that *G. alba* is similarly constrained, in that current local conditions are close to or exceed their physiological limits, with limited cooler–wetter environmental space to move into, and the environmental matrix between populations being unfavourable for dispersal. Hence, they are highly vulnerable to further climate change, and this may be true for other amphibian habitat specialists that rely on seepages or intermittent or isolated drainage systems, with limited ability to disperse in larval, juvenile or adult life stages. Headwater systems and seasonal streams are expected to be particularly altered by drying climates, as their hydrology and hydroperiod are highly affected by seasonal and annual weather patterns (Brooks, 2009). A number of threatened amphibians in Australia are restricted to small areas associated with seepages or seasonal streams (e.g. *Philoria frosti*, *Pseudophryne corroboree*, *Pseudophryne pengilleyi*, *Taudactylus pleione*) and may be similarly at high risk from hydrological changes associated with a drying climate. Furthermore, those species that rely on seasonal streams, or seepages in mid-latitude areas experiencing significant drying, may already be experiencing niche contraction from climate change (Araújo *et al.*, 2006; Scheele *et al.*, 2012).

Species with limited dispersal capability, such as *G. alba*, may be dependent on behavioural and/or genetic adaptation to respond to climatic changes. In our study, we did not account for acclimation or behavioural plasticity, which could reduce extinction risk under climate change (Ruiz-Aravena *et al.*, 2014; Riddell *et al.*, 2018). For example, acclimation can increase the *CT_max_* of amphibians by up to 4°C (Brattstrom, 1968), but in general, amphibians are thought to have a limited capacity for thermal acclimation (Niehaus *et al.*, 2012; Enriquez-Urzelai *et al.*, 2019; Morley *et al.*, 2019) and plasticity has been documented more often at colder (*CT_min_*) than warmer (*CT_max_*) extremes (John-Alder *et al.*, 1988; Oyamaguchi *et al.*, 2018). There has been less research into the potential for amphibians to adapt to increasing water stress, but terrestrial-breeding species can exhibit intraspecific variation in desiccation tolerance (Rudin-Bitterli *et al.*, 2020) and genomic analysis has revealed local adaptation to environmental regimes (Cummins *et al.*, 2019). The rapid loss of *G. alba* populations, associated with a drying microclimate, suggests that the species is not adapting to changes fast enough to prevent localized extinctions from a large portion of their range.

### Water may be more limiting for amphibians in drying climates

Studies that have utilized physiological traits to investigate the vulnerability of amphibian species to future climate changes often focus solely on thermal traits and expected temperature changes (e.g. Gerick *et al.*, 2014; Oyamaguchi *et al.*, 2018; von May *et al.*, 2019). However, we found that current extremes in temperature appear to be less of a threat in riparian frog habitats compared to extremes in soil dryness. During a dry year, most *G. alba* sites experienced prolonged conditions likely to cause hydric stress (average 59 days), whereas temperatures regularly surpassed thermal thresholds (*T_opt_*) at only one site. Water loss can be more limiting than extreme temperatures in some amphibian species and in drier habitats that experience marked seasonality (Tracy *et al.*, 2013; Peterman and Semlitsch, 2014). Water loss may be a particular constraint for amphibians that depend upon soil-bound water to maintain their water balance, rather than those species that can readily exploit unbound water (e.g. in ponds, seepages, streams and on the vegetation/litter surface). Further, hydration and temperature stress interact strongly, and considering either on their own may severely underestimate the impacts of climate change (Rohr and Palmer, 2013; Lertzman-Lepofsky *et al.*, 2020; Greenberg and Palen, 2021).

Thermoregulation via evaporative cooling often comes as a trade-off with water loss, and consequently, frogs may risk desiccation in hot conditions, rather than avoid sub-optimal temperatures (Tracy *et al.*, 2008; Beltrán *et al.*, 2019). Similarly, as individuals dehydrate, their performance and thermal tolerances may be reduced (Anderson and Andrade 2017), and their potential for successful reproduction can decline in concert (Mitchell, 2001b). Therefore, water loss leading to desiccation may be more immediately limiting than high temperatures for many amphibians in a changing climate, and it is hence necessary to consider not only how close species are to their thermal optima and tolerance limits, but also their hydric limits, when modelling responses to environmental change (Bovo *et al.*, 2018).

## Funding

This work was supported by the Australian Government’s National Environmental Science Program through the Threatened Species Recovery Hub; the Forrest Research Foundation; the Mohamed bin Zayed Species Conservation Fund; the Holsworth Wildlife Research Endowment; and The Ecological Society of Australia.

## Supplementary Material

Frog_Ecophysiology_Supp_Material_FINAL_coab027
