# Supplementary material for: Low desiccation and thermal tolerance constrains a terrestrial amphibian to a rare and disappearing microclimate niche
Source: Conserv Physiol. 2021 Apr 28;9(1):coab027. doi: 10.1093/conphys/coab027 (PMC8084025; doi:10.1093/conphys/coab027)
Supplement: Frog_Ecophysiology_Supp_Material_FINAL_coab027 [file frog_ecophysiology_supp_material_final_coab027.docx]

**Supplementary Material Conservation Physiology**

**Low desiccation and thermal tolerance constrains a terrestrial amphibian to a rare and disappearing microclimate niche**

Emily P. Hoffmann^a, b^, Karen L. Cavanough^c^ and Nicola J. Mitchell^b^

^a^corresponding author; emily.hoffmann@research.uwa.edu.au; +61-474-797-277

^b^School of Biological Sciences, The University of Western Australia, 35 Stirling Highway, Crawley, Western Australia 6009, Australia

^C^ Perth Zoo, Department of Biodiversity, Conservation and Attractions, P.O. Box 489, South Perth, Western Australia 6951, Australia

1. ***Incubation temperature treatments***
2. ***Supplementary Methods 1***

*Development rate function parameters*

1. ***Water loss and microclimates across riparian and terrestrial habitats***
2. ***Supplementary Methods 2***

*Water retention curves for soils from Geocrinia habitats*

1. ***Microclimates in frog habitats over time* *and physiological tolerances***
2. ***Trends in the recent climate***

***Incubation temperature treatments***

**Table S1.** Incubation temperature treatments and mean development times (stage 19-39; Anstis 2010) of *Geocrinia alba* and *G. vitellina* larvae.

| **Species** | **Treatment (**°C**)** | **Mean temperature (**°C**)** | **Development time (days)** | **SD** | ***n* split-clutches (individuals)** |
| --- | --- | --- | --- | --- | --- |
| *G alba* | 15 | 15.6 | 62.5 | 0.55 | 6 (27) |
|  | 18 | 17.8 | 48.0 | 0.15 | 6 (27) |
|  | 21 | 20.9 | 36.0 | 0.32 | 6 (24) |
|  | 21 (19-23.5) | 20.9 | 37.3 | 0.34 | 6 (26) |
| *G. vitellina* | 15^ | 15.0 | 68.6 |  |  |
|  | 18 | 17.8 | 48.7 | 2.88 | 6 (40) |
|  | 20 | 19.5 | 41.5 | 0.50 | 6 (23) |
|  | 21 (19-23.5) | 21.0 | 36.5 | 0.05 | 6 (38) |
|  | 25 → 21* | 20.4 | 36.4 | 0.30 | 5 (11) |
|  | 25 (20-30) → 21 (19-23.5)* | 20.7 | 35.3 | 0.28 | 3 (7) |

*^Development time from Mitchell 2001 *treatment regime temperature lowered after initial 5 days*

**Figure S1.** Schematic of the daily constant and fluctuating incubation regimes for *G. alba* and *G. vitellina.* Arrows denote two *G. vitellina* treatments (25 °C and 25 ± 5 °C, dashed lines) that were adjusted to lower temperatures after five days due to high mortality.

***Supplementary Methods 1***

*Development rate function parameters*

The non-linear development rate function is defined by five parameters:

*ra* = *b*110−*v*2(1−*b*5+*b*5*v*2)

*u* = (*T*−*b*3) ⁄(*b*3−*b*2) − *c*1

*v* = (*u*+*eb*4*u*)⁄*c*2

*c*1 = 1⁄(1+.28*b*4+.72ln(1+*b*4))

*c_2_* = 1+*b*4 ⁄(1+1.5*b*4 +.39*b*42)

where *ra* is the development rate (percentage per day); *b*1 is the maximum development rate; *b*2 is approximately the temperature (<*b*3) at which *ra* falls to *b*1⁄10; *b*3 is the approximate temperature at which *ra* is maximum; *b*4 determines how sharply *ra* approaches 0 at high temperatures; and *b*5 controls the asymmetry of *ra*. The terms *c*1 and *c*2 allow the approximate interpretations of *b*2 and *b*3 given above, and *T* is the temperature (Dallwitz and Higgins, 1992a).

***Water loss and microclimates across riparian and terrestrial habitats***

**Figure S2.** Dehydration rates (rates of water loss) of *G. alba, G. vitellina* and large agar frog models from 100% to 90% of their initial body mass. Experiments were conducted in a desiccation chamber at 18 °C, 20-30% humidity.

**Figure S3.** Comparison of daytime thermal microclimates available within *G. alba* sites in riparian habitats. Lines show mean (*n =* 5) temperatures under logs, moss, litter, sedge and with no cover (open), as well as the air temperature and soil temperature recorded at one site (GA37C), indicating that soil temperatures buffer ambient temperatures in riparian habitat. All data were recorded on February 3 2020, which was a hot and dry sunny day (maximum air temperature 29.9 °C) that coincided with the agar frog model experiments.

**Figure S4.** Daytime water loss (±SE) of frog models and average maximum temperatures under different microhabitat cover types in *Geocrinia* *alba* habitats.

**Table S2.** Results of statistical analyses (multivariate ANOVA or t-test) on the influence of a series of variables on water loss, temperature and soil moisture in different habitat types (top rows) and groundcover types (bottom rows).

| **Water Loss** | | | | | **Temperature** | | | | | **Soil Moisture (VWC)** | | | |
| --- | --- | --- | --- | --- | --- | --- | --- | --- | --- | --- | --- | --- | --- |
| Habitat type | | | | | | | | | | | | | |
|  | *df* | *F* | *p* |  |  | *df* | *F* | *p* |  |  | *df* | *F* | *p* |
| Habitat | 2,79 | 37.39 | **<0.001** |  | Habitat | 2,83 | 49.99 | **<0.001** |  | Habitat | 2,83 | 59.69 | **<0.001** |
| Season | 1,79 | 54.40 | **<0.001** |  | Season | 1,83 | 198.93 | **<0.001** |  | Season | 1,83 | 0.03 | 0.871 |
| Size | 1,79 | 7.54 | **0.008** |  | Habitat:Season | 2,83 | 11.44 | **<0.001** |  | Habitat:Season | 2,83 | 0.76 | 0.473 |
| Habitat:Season | 2,79 | 11.91 | **<0.001** |  |  |  |  |  |  |  |  |  |  |
| Habitat:Size | 2,79 | 1.28 | 0.283 |  |  |  |  |  |  |  |  |  |  |
|  |  |  |  |  |  |  |  |  |  |  |  |  |  |
| Ground cover type | | | | | | | | | | | | | |
|  | *df* | *F* | *p* |  |  | *df* | *F* | *p* |  |  | *df* | *t* | *p* |
| Cover type | 4,76 | 26.32 | **<0.001** |  | Cover type | 4,84 | 22.93 | **<0.001** |  | Season | 7.89 | -0.70 | 0.500 |
| Season | 1,76 | 23.41 | **<0.001** |  | Season | 1,84 | 39.70 | **<0.001** |  |  |  |  |  |
| Size | 1,76 | 3.41 | 0.069 |  | Season:Covertype | 4,84 | 1.94 | 0.111 |  |  |  |  |  |
| Season:Covertype | 4,76 | 6.18 | **<0.001** |  |  |  |  |  |  |  |  |  |  |
| Size:Covertype | 4,76 | 2.26 | 0.070 |  |  |  |  |  |  |  |  |  |  |
|  |  |  |  |  |  |  |  |  |  |  |  |  |  |

***Supplementary Methods 2***

*Water retention curves for soils from Geocrinia habitats*

To determine the relationship between soil water potential and volumetric water content (aka soil moisture) we established water retention curves for a range of soil types found across *G. alba* habitats. Soil water content is easy and quick to measure in the field but the water available to plants/animals depends on soil water potential, which is independent of soil physical properties. Therefore, developing water retention curves for specific soils is useful for converting field measurements of soil water content into water potential, so that the hydric thresholds of a species can be interpreted in the same units of pressure (kPa).

Riparian soils collected across range *of G. alba* (*n* = 41), *G. vitellina* (*n =* 19) sites were categorised into five texture categories ranging from the sandiest to clayiest soils (Hoffmann et al. 2020). Three replicate samples of each soil type were air dried and then packed inside a PVC ring sitting on top of filter paper on saturated ceramic plates. Soils were allowed to saturate for at least 24 hours, and plates were then placed and sealed inside pressure plate extractors set to pressures ranging from -10 kPa to -1500 kPa, and allowed to equilibrate (as gauged when the outflow became static). Soil water content of each sample was measured immediately after plates were depressurised by weighing equilibrated wet soil samples, and then oven drying the soil at 105 °C for 24 hours before reweighing. Volumetric water content was averaged for each soil type for each treatment, and plotted against the corresponding pressure (Figure A5).

**Figure S5.** Water retention curves for soil from *Geocrinia alba* and *Geocrinia vitellina* habitats. Each line represents one of the five defined/categorised soil textures/types ranging from the sandiest to clayiest soils. Sandier soils hold less water than clayier soils at the equivalent water potential.

***Microclimates in frog habitats over time* *and physiological tolerances***

Table S3. Summary of soil microclimates in frog habitats from July 2018 to June 2020 and number of days physiological tolerances were exceeded.

|  | | **Temperature (°C)** | | | | | **Water potential (-kPa)** | | |
| --- | --- | --- | --- | --- | --- | --- | --- | --- | --- |
|  |  |  | **2018-19** | | **2019-20** | |  | **2018-19** | **2019-20** |
| **Site** | **Estimated popn size*** | **Maximum** | **Days >23.3** | **Days >29.6** | **Days >23.3** | **Days >29.6** | **Minimum** | **Days < -50** | **Days < -50** |
| GA47G | 42 | 21.1 | 0 | 0 | 0 | 0 | 580.6 | 0 | 49 |
| GA37C | 22 | 22.3 | 0 | 0 | 0 | 0 | 248.5 | 0 | 37 |
| GA35B | 10 | 24.0 | 0 | 0 | 3 | 0 | 643.8 | 0 | 76 |
| GA42B | 100 | 22.1 |  |  | 0 | 0 | 34.0 |  | 0 |
| GA2B | 10 | 26.6 |  |  | 17 | 0 | 1456.8 |  | 120 |
| GA6E | 22 | 22.4 | 0 | 0 | 0 | 0 | 317.8 | 0 | 27 |
| GA24A | 10 | 30.0 | 29 | 0 | 86 | 2 | 974.3 | 15 | 43 |
| GA37E | 100 | 21.2 | 0 | 0 | 0 | 0 | 10.2 | 0 | 0 |

** Based on 2017-2018 estimates of calling males (see Hoffmann et al.,2020) and assumed 50-50 sex ratio (see Driscoll 1996).*

***Trends in the recent climate***

**Table S4.** Recent climate trends of rainfall, maximum air temperatures and streamflow from weather stations located near *G. alba* sites.

| **Climate measure** | **Weather station**  **(source)** | **Location (and distance) to nearest extant *G. alba* population** | **Period** | **Sen’s slope/ Trend**  **(per decade)** | **Tau & Significance** |
| --- | --- | --- | --- | --- | --- |
| **Annual rainfall** | Forest Grove  (BOM) | -34.0744 115.0808  (3.4 km) | 1990-2019 (last 30 years) | -10.89 mm yr^-1^  (-108.94 mm decade^-1^) | tau = -0.319  *p* = 0.031 |
| **Annual mean maximum temperature** | Cape Leeuwin  (BOM) | -34.3728 115.1358  (25.2 km) | 1990-2019 (last 30 years) | + 0.013 °C  (+0.13°C decade^-1^) | tau = 0.22,  *p* =0.10118 |
| **Annual streamflow discharge ML** | Forest Grove – Chapman Brook  (WIN database, 2020) | -34.0834 115.1532  (1.6 km) | 1996-2019 | -191.355 ML year^-1^ | tau = -0.188,  *p* = 0.20586 |

BOM – Bureau of Meteorology, WIN – Water Information Network.

**Table S5.** Comparison of annual rainfall, maximum air temperatures and streamflow in 2018-2019 with the long-term average (1961-90)* from weather stations located near *G. alba* sites.

| **Climate measure** | **Long term average (period)** | **2018 anomaly** | **2019 anomaly** |
| --- | --- | --- | --- |
| **Rainfall** | 1154.3 mm (1961-1990) | -155.9 mm | -284.1 mm |
| **Streamflow** | 12,521 ML (1995-2019)* | -123.0 ML yr^-1^ | -5956. ML yr^-1^ |
| **Temperature** | 19.88 °C (1961-1990) | +0.22 °C (1961-90) | +0.41 °C (1961-90) |

*streamflow data were not available from 1961, so in 2018 and 2019 the difference from the mean of all available data are reported, rather than an anomaly.

**Figures S6.** Recent rainfall (top) maximum air temperature (bottom) trends in the southwest of Western Australia. Rainfall trends are based on Forest Grove weather station data (1926-2019) and air temperature trends are based on Cape Leeuwin weather station data (1897-2018). Bars represent normalised anomalies from the standard averages based on the 1961-1990 climate. The black line indicates the linear trend over the last 30 years. Arrows pointing to the last two years show the years of this study.
